# An Electronic Nose for Reliable Measurement and Correct Classification of Beverages

**DOI:** 10.3390/s110606435

**Published:** 2011-06-17

**Authors:** Mazlina Mamat, Salina Abdul Samad, Mahammad A. Hannan

**Affiliations:** 1 Institute of Microengineering and Nanotechnology, Universiti Kebangsaan Malaysia, 43600 UKM Bangi Selangor, Malaysia; E-Mail: mazlina@eng.ukm.my; 2 Faculty of Engineering and Built Environment, Universiti Kebangsaan Malaysia, 43600 UKM Bangi Selangor, Malaysia; E-Mail: salina@eng.ukm.my

**Keywords:** electronic nose design, beverage classification, principal component analysis, multi layer perception

## Abstract

This paper reports the design of an electronic nose (E-nose) prototype for reliable measurement and correct classification of beverages. The prototype was developed and fabricated in the laboratory using commercially available metal oxide gas sensors and a temperature sensor. The repeatability, reproducibility and discriminative ability of the developed E-nose prototype were tested on odors emanating from different beverages such as blackcurrant juice, mango juice and orange juice, respectively. Repeated measurements of three beverages showed very high correlation (*r* > 0.97) between the same beverages to verify the repeatability. The prototype also produced highly correlated patterns (*r* > 0.97) in the measurement of beverages using different sensor batches to verify its reproducibility. The E-nose prototype also possessed good discriminative ability whereby it was able to produce different patterns for different beverages, different milk heat treatments (ultra high temperature, pasteurization) and fresh and spoiled milks. The discriminative ability of the E-nose was evaluated using Principal Component Analysis and a Multi Layer Perception Neural Network, with both methods showing good classification results.

## 1. Introduction

The electronic nose (E-nose) has been tested in many fields where odors and gases play a role. Its applicability as been tested in such diverse fields as the food industry, environmental studies, the automotive industry, microbiology and so on. E-noses have been used to monitor cultivation processes [1], and indoor air quality [2,3], to discriminate between polymer samples as a means to reduce the unpleasant odor of new cars in the automotive industry [4], to monitor volatile compounds in the effluent of a domestic wastewater treatment plant [5] and many more applications. However, in recent times more published literature has described the use of E-noses in food analysis, which includes process monitoring, shelf life investigation, freshness evaluation, authenticity assessment and other quality control studies [6]. The application of E-noses in food analysis is due to their high sensitivity and strong data correlation with conventional methods such as human sensory evaluation, as well as the fact they offer shorter analysis times, lower costs, non-destructive testing and also the fact that the tests can be automated.

The majority of the work on E-noses in food analyses was conducted using commercially available sensing systems. Discussions on commercially available E-nose models such as FOX (Alpha Mos) and Cyranose and Znose and their applications in a variety of food analyses can be found in [7–13]. Despite the numerous E-nose selections commercially available in the market, most researchers are still fabricating their own E-nose prototypes. This is mainly due to the high price of commercial E-noses and the limitations of their methods. For example, E-noses vary in price from US $ 10,000 to US $ 33,300. Besides their higher prices, commercial E-noses come equipped with specific sampling procedures, tools and accessories are sometimes not suitable for a particular research objective. Therefore, by fabricating their own E-nose prototypes, researchers have full control over the design, including sensor selection, sampling procedures, data acquisition, control and analysis, respectively.

Efforts to fabricate E-noses can be found in many literatures. Most were developed using metal oxide sensors from Figaro Gas Sensors (Osaka, Japan). This is due to these sensors’ features, which are low cost, long life, high sensitivity and the fact they only require simple heating and measuring circuits. El Babri *et al.* [14] developed an E-nose system consisting of a sensor array, sensor cell, sampling vessel and measurement rig and data acquisition system. The sensors array comprised six metal oxide gas sensors from Figaro Sensors—TGS823, TGS825, TGS826, TGS831, TGS832 and TGS882—with a temperature sensor and a humidity sensor. The E-nose was used to monitor the freshness over time of sardines stored at 4 °C by grouping the sardines into three categories: fresh, medium and aged. Panigrahi *et al.* [15] designed an E-nose system comprised of five components—sampling chamber, fan and pump controller/timer unit, sensor support and interface unit, power supply unit—and a data acquisition system. Seven metal oxide sensors—TGS2611.5(%1), TGS2611.5(%2), TGS2602, TGS880, TGS822, TGS812, TGS4160—and one integrated sensor sensitive to temperature and relative humidity were used. The system was employed to analyze the volatile compounds emanating from beef strip loins stored at 4 °C and 10 °C as a means to classify them into two groups (unspoiled and spoiled). Another laboratory-made E-nose was fabricated by Zhang *et al.* [16] to detect the freshness of beef over time. The developed E-nose system can be considered as a simple one consisting of a sensing chamber and data acquisition system. Six metal oxide sensors: TGS2610, TGS2600, TGS2611, TGS2620, TGS2602 and TGS2442, were used in the design. Other laboratory-fabricated E-noses can be found in Daqi *et al.* [17], Brezmes *et al.* [18] and Brudzewski *et al.* [19]. Most of the laboratory fabricated E-noses comprise eight or less gas sensors with various selectivity and various sampling procedures designed for the specific intended applications. Some have more accessories, while some have less. Regardless of the differences, all the fabricated E-noses have shown encouraging performance in the tested applications. Further study reveals that the performance of laboratory made E-noses is comparable to that of commercial E-noses available on the market. Inspired by this, we decided to develop our own E-nose for classification and regression analyses of beverage aromas. This paper describes in detail the development and presents the results of several experiments conducted to evaluate the reliability of our E-nose prototype to produce correct measurements.

## 2. Method and System

The method and system of the developed system is divided into two parts, namely the electronic nose system and the analytical tools, respectively. The details are explained below.

### 2.1. Electronic Nose System

**Figure 1.**
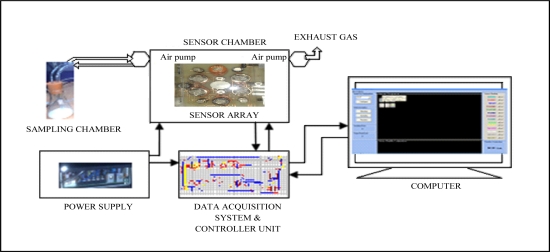
The E-nose system.

The E-nose was developed using the C++ programming language and fabricated at the Digital Signal Processing Laboratory, Universiti Kebangsaan Malaysia. The program interacted with the cleaning or sampling process activation, read and interpreted the digital values, computed the features of the sensor responses and stored the values in a file for analysis. The E-nose consists of five key components: sampling chamber, sensor chamber, data acquisition system and controller unit, power supply and graphic user interface on a computer. Figure 1 shows the block diagram of the E-nose system. Details of each of these components are explained in the following sections.

#### 2.1.1. Sampling Chamber

The sampling chamber is a 100 mL conical flask equipped with a rubber stopper. The stopper has two holes and each one is attached to a plastic tube. One of the tubes provides a route for the odor from the sampling chamber to the sensor chamber, while the other tube provides a route for the odor in the sensor chamber to the sampling chamber. To increase flow, a miniature air diaphragm pump (CTS series, 165 kPa, 508 mmHg, Hargraves Technologies Corp., Mooreville, NC, USA), is used to suck odors from the sampling chamber to the sensor chamber.

#### 2.1.2. Sensor Chamber

The sensor chamber has 8.5 cm × 12 cm × 3 cm dimensions, is airtight and was constructed from Perspex^®^ glass, which is non-reactive to chemical or food vapors. Fourteen thick film metal oxide sensors (Figaro USA, Inc.) and one temperature sensor (National Semiconductor, Santa Clara, CA, USA) were mounted on a PCB and drilled into the floor of the sampling chamber. All sensors were arranged in an oval and symmetrical form. There are no rules applied in selecting the types of sensor and quantity used [20–22]. However, sensors must be of different sensitivity to ensure the E-nose selectivity. The type of sensors used and their corresponding sensitivity to a particular gas are listed in Table 1.

**Figure 2.**
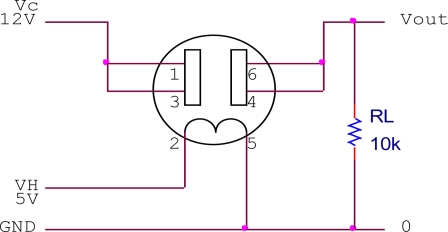
Measuring circuit for TGS8xx sensor.

**Figure 3.**
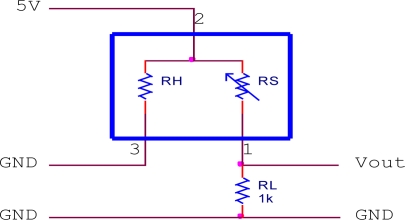
Measuring cicuit for TGS2xxx sensor.

**Figure 4.**
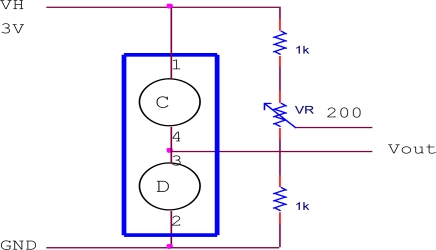
Measuring circuit for TGS6812 sensor.

The Figaro sensors require a simple heating and measuring circuit to work. The heating circuit was built inside the sensor and needs to be heated to certain temperature (40 °C) by providing a constant dc voltage. At an elevated temperature, the sensor is expected to be sensitive to a particular gas present in the air. In the presence of a detectable gas, the sensor conductivity increases and the measuring circuit converts the change in conductivity to a voltage signal. Figures 2 to 4 show the measuring circuits for the TGS8xx, TGS2xxx and TGS6812 sensors, respectively. The TGS8xx sensor requires two voltage inputs to operate: the 5 V heater voltage (V_H_) is required to stabilize the sensing circuit inside the sensor while the 12 V circuit voltage (V_C_) is required for measuring the sensor output. The sensor’s output response measured in voltage (V_out_) can be measured across the 10 kΩ load resistor (RL). The TGS2xxx sensors require one voltage input (5 V) for both V_H_ and V_C_ voltage by connecting 1 kΩ load resistor, the sensor response can be measured across it. For the TGS6812 sensor, a LM1117T voltage regulator is used to provide 3 V heater voltage by regulating the 5 V voltage source. A 200 Ω variable resistor was adjusted to certain resistance by observing the V_out_ of the sensor in the laboratory air condition which is free of combustible gases. The miniature air diaphragm pump was mounted on the outside of the sensor chamber and operates to clean the sensor chamber by sucking out the odors from the sensor chamber during the cleaning cycle.

#### 2.1.3. Data Acquisition and Controller Unit

**Figure 5.**
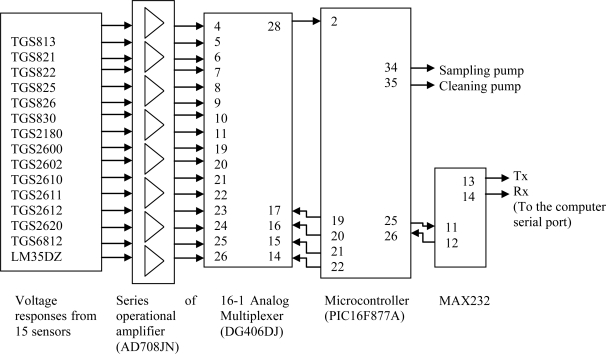
The block diagram of the developed Data Acquisition and Controller Unit.

**Figure 6.**
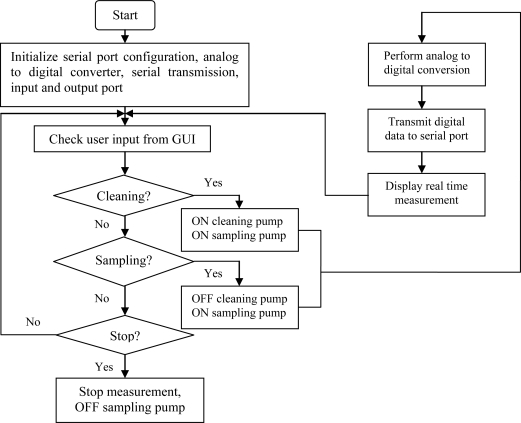
The flowchart of the program embedded into the PIC16F877a microcontroller.

The data acquisition and controller (DAC) unit consists of AD708JN amplifiers, DG406DJ analog multiplexer, PIC16F877A microcontroller and MAX232 for serial interfacing. Voltage output from sensor is connected to the AD708JN operational amplifier. The gain of the operational amplifier was determined on a one to one basis, depending on the strength of each sensor’s response. The amplified voltage signals of the 14 sensors were connected to the DG406DJ analog multiplexer and the output of the analog multiplexer was connected to channel 0 of the PIC16F877A microcontroller. A program to read, perform analog to digital conversion, receive and transmit data was written in assembly language (MPLAB IDE, version 7.20) and embedded into the PIC16F877A microcontroller. Figure 5 shows the block diagram of the DAC and Figure 6 shows the flowchart of the program embedded into the microcontroller.

#### 2.1.4. Measurement Protocol

The measurement starts by first performing the baseline correction of the sensors by purging the sensors with ambient air for 200 s. Ambient air without any pretreatment was used as a means to reduce the accessories of the e-nose system. From observations, the use of ambient air is sufficient to completely remove odors from the sensor chamber, hence producing a steady baseline. This ensures that the sensors are completely free from possible contaminant odors from previous measurements. Then the sampling chamber which contains the sample was attached to the sensor chamber and the odor was sucked into the airtight sensor chamber for 200 s. When the sampling period was over, the sensor chamber was cleaned again for another 200 s by purging ambient air into the sensor chamber. The cleaning and sampling time of the measurement process were determined based on the obtained sensor responses that have been tested. It was found that 200 s cleaning time and 200 s sampling time were sufficient to clean and to sample the beverages’ odor. Thus, the total time required for the cleaning and sampling process are 10 min (600 s = 200 s + 200 s + 200 s). In the process, the voltage reading of the sensors were also acquired and saved. During measurement, the temperature of the sensor chamber was monitored at 40 °C. The E-nose was set to repeatedly measure odors emanating from three different beverages. The beverages used were blackcurrant juice, mango juice and orange juice, all products of a local Malaysian food manufacturer. In this experiment, 50 mL of juice were used and 10 measurements were conducted for each juice. The measurements were conducted according to the described protocol.

### 2.2. Analytical Tools

The following sections describe the analytical analyses conducted on the developed E-nose. The analyses include the repeatability and reproducibility tests and also the discrimination tests of the developed E-nose.

#### 2.2.1. Repeatability and Reproducibility

As analytical instruments, E-noses must have high repeatability and reproducibility. Repeatability is defined as the ability to produce the same pattern for a sample on the same array over short intervals of time, while reproducibility can be defined as the ability of different sensor batches or different instruments to produce the same pattern for the same sample [23]. The repeatability and reproducibility of the E-nose are assessed by calculating the correlation coefficients of the features from one measurement to the other measurements of the same sample.

#### 2.2.2. Discriminative Ability

In many applications, the E-nose is coupled with pattern recognition algorithms to solve classification problems. The idea behind E-nose-based classification relies on the ability of the gas sensors to produce dissimilar patterns for different substances. To investigate the ability of the developed E-nose to discriminate different substances, three experiments were conducted. The first experiment was conducted to investigate the ability of the E-nose to discriminate between five different beverages: blackcurrant juice, orange juice, mango juice, soy milk and fresh milk. For each beverage, measurements were conducted on different packs of the same product. Altogether 51 data sets were collected which consisted of the measurements of 12 blackcurrant juice, 10 mango juice, 10 orange juice, 10 soy milk and nine fresh milk samples. The second experiment was conducted to investigate the ability of the E-nose to differentiate between UHT and pasteurized milk. For this experiment, 18 packs of UHT milk and 22 packs of pasteurized milk were used. The third experiment was conducted to investigate the ability of the E-nose to differentiate between fresh and spoiled milk. Twenty three packets of milk were used in the experiment. The measurement of fresh milk was carried out during the first hour of opening while the measurement of spoiled milk was carried out 24 hours after opening under room temperature storage conditions. These measurements resulted in 23 fresh milk data and 23 spoiled milk data points. In all experiments, 25 mL of sample were used and all samples were at ambient temperature during measurement. To analyze the patterns produced by the E-nose, two pattern recognition algorithms: unsupervised Principal Component Analysis (PCA) and supervised Multi Layer Perception (MLP) neural network were used. These analyses were conducted using the stats tools in Matlab 2007a.

##### Principal Component Analysis

The PCA is a common pattern recognition algorithm used to analyze data obtained from E-nose systems [24,25]. In particular, PCA is used to reduce the complexity of data (features of the E-nose responses) by computing a new, much smaller set of uncorrelated variables which best represent the original data. This is done by projecting the high dimensional data set in a dimensional reduced space based on the uncorrelated and orthogonal eigenvectors of the covariance matrix computed from the E-nose features. These eigenvectors were called principal components (PC) of the features and were arranged in sequence where the first principal component was the one with the greatest amount of variance, followed by the second greatest and so on. The plot of the original data in the new space defined by the first few principal components will give visual interpretation on how the original data are scattered. In particular, the plot will shows features that have small variation appear together while the features with large variation appear distant. Therefore PCA is able to expose some clusters of the data naturally. The PCA is a straightforward analysis, which is validated through conducting several experiments, measurements and testing the data.

##### Multi-Layer Perception Neural Network

The most popular ANN used in classification is Multilayered Feed Forward (MLP) neural network trained by Back-Propagation (BP) algorithm. As the name implies, the MLP has many layers consisting of one input layer, one or more hidden layers and one output layer. Each layer has its own neurons, where neurons in the same layer are not connected with each other but all the neurons of one layer feed the neurons of the following layer. Details regarding MLP and the BP algorithm can be found elsewhere [26]. The MLP trained by BP algorithm is a supervised ANN, meaning that it requires training by using a set of known data (training data) systematically until the error between real and generated target is minimized. Throughout the learning process, MLP will update its weights or parameters according to the input-target specified by the training data. The updated weights or parameters generated in the learning phase are used to classify new data accordingly.

## 3. Results and Analytical Discussion

### 3.1. Performance Evaluation on Feature Extraction and Pre-Processing

**Figure 7.**
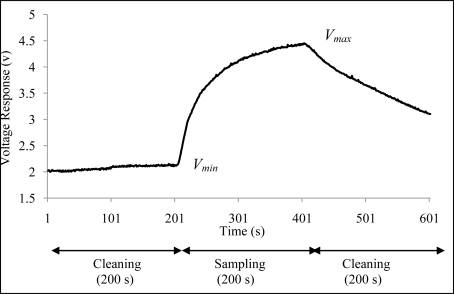
The typical orange juice voltage response captured by the TGS822.

Figure 7 shows a voltage response obtained by the TGS822 from the measurement of orange juice. From the voltage response, the average voltage value at 380 s to 400 s (*V_max_*) and the average voltage value at 180 s to 200 s (*V_min_*) were computed. From these values, the relative response of the sensor given by *Vr = (V_max_* − *V_min_)/V_min_* was calculated. The *Vr* values obtained from 14 sensors were called features and were used in the analysis to investigate the repeatability, reproducibility and discriminative ability of the developed E-nose.

### 3.2. Repeatative and Reproductive Analysis

The voltage responses of 14 sensors were obtained from repeated measurements on the same sample and different samples. This observation was supported by the correlation coefficient computed on the feature series for the three beverages as presented in Table 2. Based on the correlation coefficients tabulated in Table 2, it can be concluded that the developed E-nose possesses very high repeatability characteristics. This was proved by the correlation coefficient of the features which scores nearly 1 for all measurements conducted on the three beverages. The correlation coefficients computed between blackcurrant and mango, blackcurrant and orange and mango and orange are 0.032, 0.376 and 0.908, respectively. These values indicate that the pattern of blackcurrant is obviously different from orange and mango, while the pattern of mango and orange are slightly comparable.

The correlation coefficient is further computed to evaluate the repeatability characteristics as shown in Tables 3 and 4, respectively. The first computation is the correlation coefficients of the measurements to their average value (MA) and the second computation is the relative standard deviation (RSD) of each sensor and the average of them. The correlation coefficients obtained by 10 measurements of three different beverages were above 0.92. This observation proved that the correlation coefficient of the features showed very high repeatability and reproducibility characteristics for the three beverages. In the RSD computation, it was observed that each sensor has different RSD value varies from 1.7 to 20.9 for the three beverages. The average RSD values for 10 repeated measurements of the three beverages are 6.97 (blackcurrant), 11.04 (mango) and 12.40 (orange), respectively. The small RSD values indicate that the sensors show relatively good precision, hence confirm the repeatability of the measurements. However, it is assumed that the 0 responses of the sensors have no significant contribution to the odor patterns of the beverage. Thus, 0 responses sensors were excluded from computing the average RSD for the 10 measurements of each beverage.

**Figure 8.**
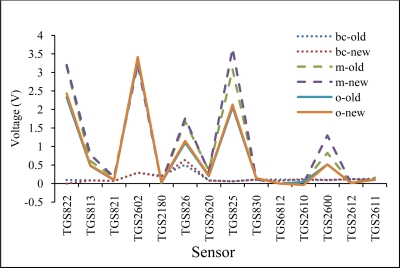
The *Vr* obtained with old and new sensor batches.

To investigate the reproducibility of the developed E-nose, all 14 sensors were replaced by a new batch and their ability to measure odor emanating from fruit juices were investigated. For this experiment, 50 mL of blackcurrant, mango and orange juices were used and one measurement was conducted using each of the two different sensor batches for the three juices. Figure 8 displays the features obtained by the E-noses using old and new sensor batches for blackcurrant (bc), mango (m) and orange (o). The features obtained by all 14 sensors show repetatability for the different juices, with mango and orange emanating strong aromas while blackcurrant emanated a weak aroma, respectively. The plots show that the developed E-nose has good reproducibility with good correlation coefficient scores for the old and new sensor batchs (r = 0.976 for blackcurrant, r = 0.990 for mango and r = 0.999 for orange).

### 3.3. Analysis of Discriminating Ability of the E-Nose by PCA

**Figure 9.**
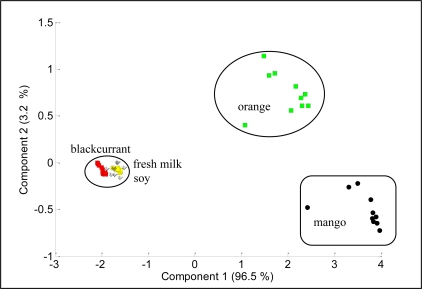
PCA plot of five different beverages.

**Figure 10.**
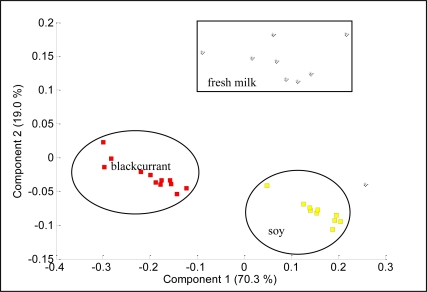
PCA plot of blackcurrant juice, soy milk and fresh milk.

**Figure 11.**
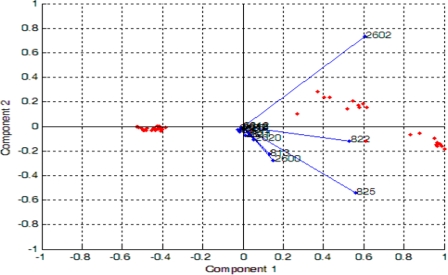
The loading values for analysis of the five beverages.

Figure 9 shows the projections of PCA results on the analysis of blackcurrant juice, mango juice, orange juice, soy milk and fresh milk. The data were plotted in a two dimensional plane formed by the first two PCs, that is PC1 and PC2, which captured 99.7% of the data variance. Samples were grouped together into three clusters, where orange and mango are clearly separated from blackcurrant, fresh milk and soy. This means that blackcurrant, fresh milk and soy share relatively comparable patterns and have totally different patterns than orange and mango. Further analysis reveals that orange and mango emanated strong aromas (high voltage responses) while blackcurrant, fresh milk and soy emanated weak aromas (low voltage response). Therefore the high voltage responses captured by orange and mango dominate the PCA of the five beverages, making the weak aroma beverages appear together and strong aroma beverages appear apart. To prove this, we excluded orange and mango from the data set and perform PCA on the blackcurrant, fresh milk and soy data only. Figure 10 shows the results indicating that the PC1 and PC2 are able to clearly separate blackcurrant, fresh milk and soy data. However, the loading values of individual sensors such as TGS2602, TGS822, TGS825, TGS2600, TGS813 and TGS2620 have a significant contribution to the discrimination of the five beverages, as shown in Figure 11.

**Figure 12.**
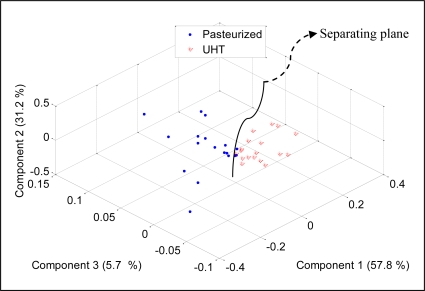
PCA plot of UHT and pasteurized milk.

To check the discriminative ability of the developed E-nose to separate between two different milk heat treatments (UHT, pasteurization), the PCA was performed and the results were projected on three most important principal components, which covered 94.7% of data variation. Figure 12 shows the score plot of the data.

**Figure 13.**
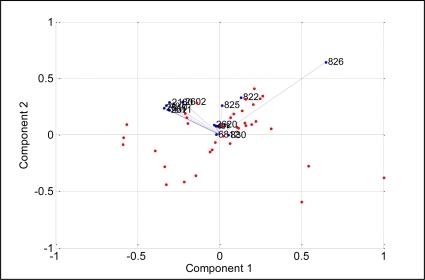
The loading values for analysis on pasteurized and UHT milks.

It can be noticed that the measurements appear together although the pasteurized milks were scattered on the left side of the separating line while the UHT ones were scattered on the right side of the separating line. This observation shows that the developed E-nose was able to produce different patterns for different milk heat treatments. Again, the loading values of sensors TGS826, TGS822, TGS825, TGS2602, TGS2180, TGS2600, TGS2610, TGS2612, TGS2611 and TGS2620 provide a significant contribution to the discrimination of pasteurized and UHT milks, respectively, as shown in Figure 13.

**Figure 14.**
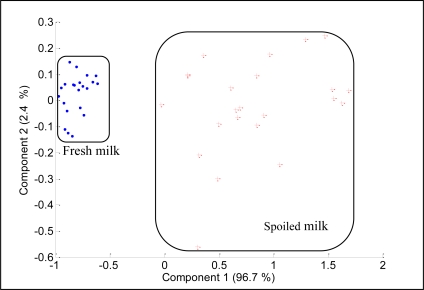
The PCA plot of fresh and spoiled milk.

The PCA of the fresh and spoiled milks generated the results shown in Figure 14. The two most important principal components, PC1 and PC2, covered 99.1% of data variation. The score plot shows that fresh milk data appear together and are distant from the spoiled milk data, which are scattered over a wider area.

**Figure 15.**
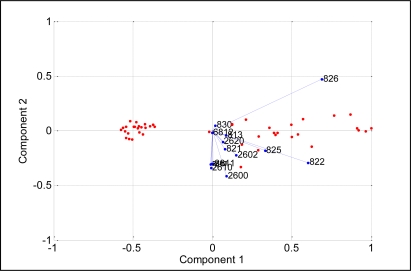
The loading values for analysis on fresh and spoiled milks.

The plots also revealed that the fresh milk and spoiled milk are linearly separable on principal component 1, hence proving that the developed E-nose is able to discriminate between fresh and spoiled milk. Again, for discrimination between fresh and spoiled milks the sensors such as TGS826, TGS822, TGS825, TGS2600 and TGS2610 play a significant role, as shown in Figure 15.

**Figure 16.**
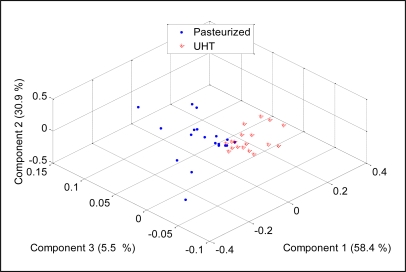
PCA plot for 14 sensors data.

**Figure 17.**
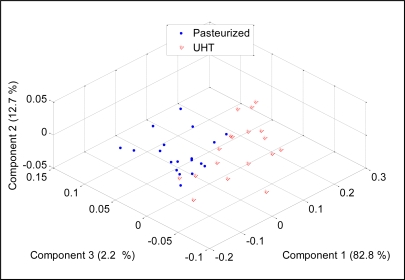
PCA plot for TGS8XX sensor.

**Figure 18.**
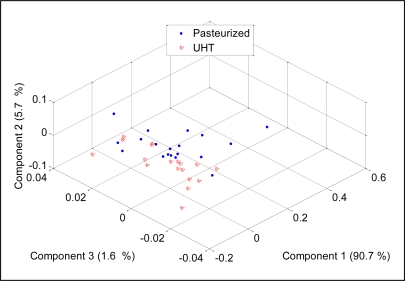
PCA plot for TGS26XX and TGS6812 sensors.

The discrimination ability of an E-nose system relies on the ability of the gas sensors to produce dissimilar patterns for different substances. Therefore the selection of sensors (sensitivity and selectivity) is an important aspect of any E-nose system. More sensors will definitely improve the E-nose in terms of odor coverage. If the E-nose is designed for a particular application, removing some sensors which show no significant response to the sample is necessary, but if the E-nose was developed as an analytical tool for various applications, the use more sensors is an advantage. For example, the PCA analysis on the data which contain measurements of pasteurized and UHT milks of all 14 sensors, only the TGS8XX sensor and only the TGS26XX and TGS 6812 sensors, respectively are shown in Figures 16 to 18.

It was observed that the 14 sensors produced the best separation between UHT and pasteurized milks. The TGS8XXs able to discriminate the milks into two groups with few measurements were mixed. The same observation was seen in PCA plot for TGS26XX and TGS6812 measurements where the milks were scattered in two areas with few measurements falling into the other area. Based on these results, we can conclude that more sensors will improve the discriminative ability of an E-nose.

### 3.4. Analysis of Discriminating Ability of the E-Nose by MLP

In all experiments, the MLP with one input layer, one hidden layer and one output layer was used. The input layer has 14 input nodes, the hidden layer has 10 hidden nodes and the output layer has one output node, respectively. Neurons in the input layer were connected to the neurons in the hidden layer via logsig function while neurons in the hidden layer were connected to neurons in the output layer using the purelin function. For the classification of five beverages, the data were divided into 26 training data and 25 testing data. The measurement data were divided randomly, whereby the first half was used as training data and the second half as testing data. The training data consisted of six blackcurrant data, five mango data, five orange data, five soy milk data and five fresh milk data, respectively, while the testing data comprised six blackcurrant data, five mango data, five orange data, five soy milk data and four fresh milk data, respectively. For the classification of UHT and pasteurized milk, 18 UHT milks and 22 pasteurized milks were divided into training and testing data. Therefore each training and testing data consist of nine UHT milks and 11 pasteurized milks, respectively. In the classification of fresh and spoiled milk, 10 fresh milks and 12 spoiled milks were used as training data while for testing data, 13 fresh milks and 11 spoiled milks were used.

The performance of MLP to classify five beverages, to differentiate between UHT and pasteurized milks and to differentiate between fresh and spoiled milks is displayed in Tables 5 to 7. The MLP is able to classify all five beverages into their respective groups with 100% accuracy. For classification of UHT and pasteurized milks, the MLP is able to recognize seven out of nine UHT milk samples correctly and able to recognize 11 pasteurized milk samples correctly with 90% accuracy. For discrimination between fresh milk and spoiled milk, the MLP is able to successfully classify all samples into their respective classes.

## 4. Conclusions

The paper has presented the design of an Electronic Nose system using 14 Metal Oxide Semiconductor gas sensors from Figaro Sensor (Japan). All the parts of the E-nose system were designed and fabricated in the laboratory. The developed E-nose has been tested to confirm its repeatability, reproducibility and discriminative ability which are important characteristics of an analytical instrument. Measurements on three beverages: blackcurrant juice, mango juice and orange juice repeatedly produced similar patterns with high correlation for the same beverage and produced different patterns with lower correlation for different beverages, consequently confirming its repeatability characteristics. The developed E-nose also produces repeatable responses in the measurement of three beverages using different sensor batches, hence confirm its reproducibility characteristics. The developed E-nose is also able to produce different patterns for different samples. The analysis using PCA and MLP on the patterns produced by the E-nose demonstrated that the E-nose has good discriminative ability, which is an important characteristic. Based on the results we concluded that the developed E-nose is a reliable analytical instrument.

## Figures and Tables

**Table 1.** List of sensors used to develop the E-nose system.

**Sensor**	**Target gas**

TGS813	Combustible Gases (methane, propane, butane)
TGS821	Hydrogen
TGS822	Organic Solvent Vapors (ethanol)
TGS825	Hydrogen Sulfide
TGS826	Ammonia
TGS830	Chlorofluorocarbons
TGS2180	Water vapor
TGS2600	Air Contaminants (hydrogen, carbon monoxide)
TGS2602	Air Contaminants (VOCs and odorous gases)
TGS2610	LP Gas and its component gases
TGS2611	Methane
TGS2612	Methane and LP Gases
TGS2620	Alcohol and Solvent Vapors
TGS6812	Hydrogen, Methane and LP Gas
LM35DZ	Temperature sensor

**Table 2.** The *r* values obtained for 10 measurements of blackcurrant, mango and orange juices.

**Beverages**	**Correlation coefficient**								

	M1M2	M2M3	M3M4	M4M5	**M5M6**	**M6M7**	**M7M8**	**M8M9**	**M9M10**

**Blackcurrant**	0.997	0.998	0.998	0.997	0.996	0.992	0.998	0.995	0.999
**Mango**	0.997	0.998	0.999	0.999	0.999	0.999	0.999	0.999	0.999
**Orange**	0.996	0.981	0.984	0.975	0.970	0.984	0.998	0.999	0.996

**Table 3.** The *r* values obtained for 10 measurements of blackcurrant, mango and orange juices.

**Beverages**	**Correlation coefficient**									

	**M1MA**	**M2MA**	**M3MA**	**M4MA**	**M5MA**	**M6MA**	**M7MA**	**M8MA**	**M9MA**	**M10MA**

**Blackcurrant**	0.992	0.991	0.998	0.982	0.988	0.960	0.980	0.991	0.982	0.928
**Mango**	0.996	0.997	0.998	0.999	0.999	0.999	0.998	0.999	0.999	0.999
**Orange**	0.997	0.997	0.997	0.999	0.989	0.999	0.999	0.998	0.998	0.998

**Table 4.** The RSD obtained for 10 measurements of blackcurrant, mango and orange juices.

**Beverages**	**Relative Standard Deviation (%)**														

	822	813	821	2,602	2,180	826	2,620	825	830	6,812	2,610	2,600	2,612	2,611	Avg.

**Blackcurrant**	12.7	4.7	0.0	4.9	5.0	6.2	0.0	6.0	8.6	0.0	0.0	0.0	0.0	7.7	**6.97**
**Mango**	1.7	11.7	18	2.3	0.0	2.3	12.8	7.1	18.3	0.0	0.0	17.3	0.0	18.9	**11.04**
**Orange**	13.4	17.0	1.3	9.6	0.0	8.3	20.9	15.5	13.9	0.0	0.0	20.9	0.0	3.23	**12.40**

**Table 5.** MLP classification results of five beverages.

**Beverages**	**Predicted as**					**Success Rate**

	**Blackcurrant**	**Mango**	**Orange**	**Soy**	**Fresh milk**	

**Blackcurrant**	6	0	0	0	0	100%
**Mango**	0	5	0	0	0	100%
**Orange**	0	0	5	0	0	100%
**Soy**	0	0	0	5	0	100%
**Fresh milk**	0	0	0	0	4	100%

**Table 6.** MLP classification results of UHT and pasteurized milks.

**Milk type**	**Predicted as**		**Success Rate**

	**UHT**	**Pasteurized**	

**UHT**	7	2	77.8%
**Pasteurized**	0	11	100%

**Table 7.** MLP classification results of fresh and spoiled milks.

**Milk type**	**Predicted as**		**Success Rate**

	**Fresh milk**	**Spoiled milk**	

**Fresh milk**	13	0	100%
**Spoiled milk**	0	11	100%
